# Novel GLP-1 Analog Supaglutide Stimulates Insulin Secretion in Mouse and Human Islet Beta-Cells and Improves Glucose Homeostasis in Diabetic Mice

**DOI:** 10.3389/fphys.2019.00930

**Published:** 2019-07-25

**Authors:** Liwei Ren, Qiaoli Cui, Wenjuan Liu, Liqian Wang, Yijing Liao, Ying Feng, Wanwan Sun, Yehong Yang, Zhaoyun Zhang, Tianru Jin, Gerald J. Prud’homme, Lina Zhang, Yiming Li, Ying Leng, Qinghua Wang

**Affiliations:** ^1^Department of Endocrinology and Metabolism, Huashan Hospital, Fudan University, Shanghai, China; ^2^Shanghai Yinuo Pharmaceutical Co., Ltd., Shanghai, China; ^3^Shanghai Institute of Materia Medica, Chinese Academy of Sciences, Shanghai, China; ^4^Division of Advanced Diagnostics, Toronto General Research Institute, University Health Network, Toronto, ON, Canada; ^5^Department of Medicine, University of Toronto, Toronto, ON, Canada; ^6^Department of Laboratory Medicine and Pathobiology, Keenan Research Centre for Biomedical Science, St. Michael’s Hospital, Toronto, ON, Canada

**Keywords:** GLP-1, supaglutide, diabetes, insulin, glucose homeostasis

## Abstract

Glucagon-like peptide-1 (GLP-1), an incretin hormone plays an important role in regulating glucose homeostasis. The therapeutic use of native GLP-1 is inadequate due to its short *in vivo* half-life. We recently developed a novel GLP-1 mimetics supaglutide, and demonstrated that this formulation retained native GLP-1 biological activities and possessed long-lasting GLP-1 actions. In this study, we further examined its abilities in regulating blood glucose in diabetic mice. We found that supaglutide stimulated insulin secretion in both mouse and human islets in a dose-dependent fashion. Oral glucose tolerance test conducted in normal ICR mice showed that supaglutide significantly decreased postprandial glucose excursions in a dose-dependent fashion. In type 2 diabetic *db/db* mice, a single-dose injection of supaglutide significantly decreased blood glucose levels, and this efficacy was lasted for at least 72 h in a dose-dependent fashion. During a 4-weeks intervention course supaglutide (twice injections per week) dose-dependently and significantly decreased fasting and random blood glucose levels in hyperglycemic *db/db* mice. Supaglutide, at a dose of 1.2 mg/kg, significantly reduced serum fructosamine levels. This was associated with significant enlargement of beta-cell mass, increased pancreatic insulin content, and increased plasma insulin level. Notably, during the intervention course supaglutide significantly reduced body-weight gain in these obese diabetic mice, associated with reduced fat mass (but not the lean mass), improved lipid profile, i.e., declined serum triglyceride, and free fatty acid levels compared to the placebo control. These finding reveals that supaglutide exerts beneficial effects in regulating blood glucose and lipid homeostasis in diabetic *db/db* mice.

## Introduction

Glucagon-like peptide 1 (GLP-1) is an incretin hormone secreted by gastrointestinal L cells in response to nutrient ingestion (Drucker and Nauck, 2006; Drucker, 2016). Owing to its attractive biological functions such as stimulation of insulin secretion in a glucose-dependent fashion, inhibiting glucagon release, suppressing appetite and slowing gastric emptying, GLP-1 is currently used as a major type of incretin-based therapy for type 2 diabetes (T2D) (Nauck et al., 1993; Wettergren et al., 1993). However, the endogenous GLP-1 is susceptible to proteolytic cleavage and rapidly degraded primarily by dipeptidyl peptidase-IV (DPP-IV), and also by rapid renal clearance, resulting in a short-circulating half-life (*t*_1/2_ < 2 min) (Deacon et al., 1995a,b). Therefore, great efforts have been made in the past decade aiming to achieve long-lasting *in vivo* GLP-1 actions. In order to fulfill this requirement, there are two strategies of GLP-1 therapy that are accessible: one is the use of DPP-IV inhibitors and the other is the development of DPP-IV-resistant GLP-1mimetics (Deacon et al., 1998; Holst and Deacon, 1998; Drucker and Nauck, 2006; Drucker et al., 2017).

Using gene engineering recombinant fusion protein techniques we developed a long-acting GLP-1 analog supaglutide by fusing GLP-1 with immunoglobulin constant region Fc fragment to form fusion protein that contains covalent binary GLP-1 molecules (Wang et al., 2010; Wan et al., 2017). We demonstrated that the fusion chimera has high avidity to the GLP-1 receptor (but not the glucagon receptor), and retains native GLP-1 biological activities (Wang et al., 2010). Our previous studies in both *in vitro* and *in vivo* settings showed that the fusion protein was relatively resistant to DPP-IV enzymatic inactivation (Wang et al., 2010), and possibly other degrading enzymes (Hupe-Sodmann et al., 1995). The long-acting efficacy of supaglutide was possibly also attributed to its enlarged molecular mass (>60 kDa) as a result of delay in clearance rate of kidney (Wang et al., 2010). In this study, we evaluated its insulinotropic effects under both *in vitro* and *in vivo* conditions using isolated mouse and human islets, and normal mice, as well as type 2 diabetic *db/db* mice.

## Materials and Methods

### Animals

Adult male C57BL/6J mice were purchased from Slack Laboratory Animal, Shanghai, China. Institute of Cancer Research(ICR)mice were purchased from Jiesijie Laboratory Animal Co., Ltd. (Shanghai, China). B6.Cg-m+/+Leprdb/J (*db/db*) mice and its lean littermates^+/+^ (from Jackson Laboratory, Bar Harbor, ME, United States) were bred in Shanghai Institute of Materia Medica (SIMM), Chinese Academy of Sciences. The animals were housed under the conditions of constant temperature (22–24°C) and a light/dark cycle of 12 h with free access to food and water. The *db/db* mice were fed with high fat diet (SLAC Laboratory Animal Co., Ltd., Shanghai, China), the lean mice and ICR mice were provided with standard chow. Animal care procedures followed the National Institute of Health Guidelines on the Care and Use of Animals and were approved by the Institutional Animal Care and Utilization Committee (IACUC), Shanghai Institute of Materia Medica, Chinese Academy of Sciences.

### Supaglutide Treatment

Supaglutide were provided by Yinnuo Pharmaceutical Technology Co., Ltd. (Shanghai, China). ICR mice were divided into four groups (*n* = 10) according to body weight. Then mice received a single subcutaneous injection with saline as control, or supaglutide (0.15, 0.3, and 0.6 mg/kg). Diabetic *db/db* mice were divided into five groups (*n* = 10, 5 males and 5 females) according to body weight and blood glucose levels. Treatment study was performed by subcutaneous injecting supaglutide (0.15, 0.3, 0.6, and 1.2 mg/kg) and saline as control twice a week for 4 weeks.

### Glucose-Stimulated Insulin Secretion (GSIS)

Mice islets were isolated from male C57BL/6J mice by collagenase digestion (Xu et al., 2006). The isolated islets were maintained in RPMI-1640 medium containing 10% FBS.

Human islets were isolated as we described previously (Purwana et al., 2014). Pancreata from cadaveric non-diabetic adult human donor (46 years old male, BMI = 26 kg/m^2^, HbA1c = 5.9%, purity > 50% as determined by dithizone staining) were retrieved after consent and human islets were isolated by the University Health Network Islet Isolation Program (Toronto, ON, Canada). The isolation protocol was reviewed and approved by the University Health Network Research Ethics Board (14-8321.2). The subject gave written informed consent in accordance with the Declaration of Helsinki. Isolated human islets were then cultured in CMRL-1066 medium supplemented with 10% FBS.

Isolated islets were cultured overnight at 37°C, then the medium was replaced with fresh Krebs-Ringer bicarbonate buffer (KRB: 115 mM NaCl, 5 mM KCl, 2.5 mM CaCl2, 1 mM MgCl2, 24 mM NaHCO3, 10 mM HEPES, 0.5% BSA; pH 7.4) supplemented with 2 mM glucose for 2 h. The isolated islets were then treated with 2 mM or 16.8 mM glucose with or without supaglutide (1 or 10 nM) in KRB buffer for 2 h. The insulin levels in the conditioned KRB buffer were measured using insulin radioimmunoassay (RIA) kits (Crystal Chem Inc., United States).

### Oral Glucose Tolerance Test (OGTT)

Mice were fasted overnight for 12 h, and provided 2.5 g glucose/kg body weight orally. Blood was drawn from the tail vein and the glucose levels were measured using an ACCU-CHEK Advantage II Glucose Monitor (Roche, IN, United States) at 0, 15, 30, 60, and 120 min after glucose administration.

### Body Composition Analysis

Bruker Minispec LF50 Whole Body Composition Analyzer, a time-domain nuclear magnetic resonance (TD-NMR) based technology was used for *in vivo* measurement of fat tissue, muscle tissue and body fluid in *db/db* mice prior to supaglutide treated and again on day 24 after drug administration according to the manufacturers’ instructions (Tinsley et al., 2004).

### Serological Study

Fructosamine (FRU) level were measured using Hitachi-7020 automatic biochemical analysis device (Hitachi, Japan). Free fatty acids (FFA) levels were determined using the kit (Wako Pure Chemical Industries, Ltd., Japan). The fasting serum triglyceride (TG), total cholesterol (TC), high-density lipoprotein (HDL), and low-density lipoprotein (LDL) levels were analyzed using commercial kits (Zhengjiang Dongou Co., Ltd., China) according to manual procedures.

### Immunohistochemistry (IHC) and Islet Beta-Cell Mass Analysis

Pancreas was performed for immunohistochemistry as described previously (Wang and Brubaker, 2002; Liu et al., 2017). The pancreas samples were fixed in 10% buffered formalin and subsequently embedded in paraffin. Each pancreatic block was serially sectioned (3 μm) throughout its length to avoid any bias from regional changes in islet distribution and islet cell composition. Then, four sections were chosen at 105 μm intervals throughout the block (every 35 sections). The sections were deparaffinized, rehydrated and placed in 3% hydrogen peroxide for 10 min at room temperature, followed by heating twice for 15 min at 90°C in a microwave, rinsing with Tris-buffered saline with Tween 80 (TBS-T) twice for 5 min. Sections were pre-processed in 5% normal goat serum for 45 min, subsequently, incubated overnight with guinea pig anti-insulin antibody (1:100) (Abcam, United States) at 4°C. Then, the sections were detected with biotinylated secondary antibodies (1:150, Abcam, United States) for 60 min. The sections were incubated with avidin–biotin–peroxidase complex (Gene Tech, China) before staining with DAB (Gene Tech, China) and subsequent hematoxylin counterstaining. Entire pancreatic slides were scanned and viewed with NanoZoomer (Hamamatsu, Hamamatsu, Shizuoka, Japan) and analyzed by using the ImageScope program (Aperio Technologies, Vista, CA, United States). Beta-cell mass (mg) was determined as pancreatic weight × (the area showing insulin positivity/total pancreatic area) as described previously (Wang and Brubaker, 2002; Liu et al., 2017).

### Measurement of Beta-Cell Proliferation and Apoptosis

Proliferative beta-cells were identified by insulin-Ki67 double immunofluorescence staining with rabbit anti-Ki67 (1:200; Thermo Fisher), guinea pig anti-insulin antibody (1:100; Abcam) and relevant secondary antibodies (1:1000; Abcam). Apoptotic beta-cells were identified by insulin and terminal deoxynucleotidyl transferase dUTP nick end labeling (Tunel) (TMR red, Roche, Mississauga, ON, Canada) dual labeling. Results are expressed as the percentage of Ki67+, or Tunel+ beta-cells.

### Statistical Analysis

All data were presented as mean ± SEM and data analysis was carried out with the Graph-Pad Prism 5 program. Data analysis was statistically performed using Student’s *t*-test or ANOVA and difference was considered as statistical significance only when *p* < 0.05.

## Results

### Supaglutide Stimulates Insulin Secretion in Mouse and Human Islets in a Dose-Dependent Fashion

**FIGURE 1:**
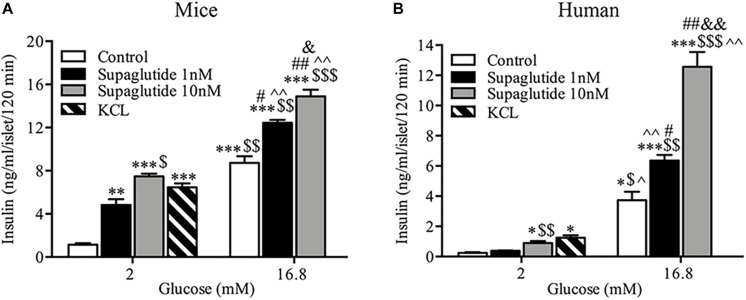
Supaglutide stimulated insulin secretion from isolated mouse and human islets. **(A)** Supaglutide dose-dependently enhances insulin secretion from isolated mouse islets. **(B)** Supaglutide dose-dependently enhances insulin secretion from isolated human islets. Data are presented as mean ± SEM, *n* = 3. ^*^P < 0.05, ^∗∗^*P* < 0.01, ^∗∗∗^*P* < 0.001, compared to control (2 mM); ^$^*P* < 0.05, ^$$^*P* < 0.01, ^$$$^*P* < 0.001, compared to 1 nM supaglutide (2 mM); ^∧^*P* < 0.05, ^∧∧^*P* < 0.01, compared to 10 nM supaglutide (2 mM); ^#^*P* < 0.05, ^##^*P* < 0.01, compared to control (16.8 mM); ^&^*P* < 0.05, ^&⁣&^*P* < 0.01, compared to 1 nM supaglutide (16.8 mM).

The microscopic images showed the quality of isolated mice islets (Supplementary Figure 1A). The islet preparation purity was examined by dithizone staining (>50%) and GSIS assays were performed to check the secretory function (Supplementary Figure 1B). Supaglutide incubation for 2 h significantly increased insulin secretion in isolated mouse islets (Figure 1A). Supaglutide at the doses tested (1 and 10 nM) increased mouse insulin secretion both at the low (2 mM) and high (16.8 mM) glucose concentrations (Figure 1A). In human islets supaglutide also remarkably increased insulin secretion (Figure 1B). However, 1 nM supaglutide (but not 10 nM) did not obviously increase insulin secretion at low glucose (2 mM) condition and the drug at both tested dose (1 and 10 nM) significantly and drastically increased insulin secretion at high glucose condition (16.8 mM) (Figure 1B). It is interesting to note, compared to its basal insulin levels, 16.8 mM glucose dramatically and significantly stimulated (∼18-fold) increase in insulin secretion, and 10 nM supaglutide in the presence of 16.8 mM glucose yielded a ∼63-fold increase in human insulin secretion compared to the basal levels.

### Supaglutide Improves Glucose Tolerance in Normal ICR Mice

**FIGURE 2:**
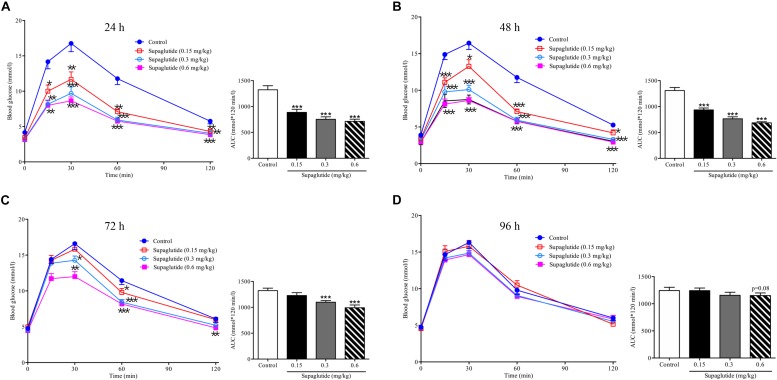
A single subcutaneous injection of supaglutide significantly and dose-dependently improves the glucose tolerance. Glucose concentrations in ICR mice undergoing an OGTT at 24 h **(A)**, 48 h **(B)**, 72 h **(C),** and 96 h **(D)** after a-single subcutaneous injection of supaglutide or vehicle. The areas under the glycaemic curves (AUC) are shown in the right panels. Data are shown as mean ± SEM. ^*^p < 0.05, ^∗∗^*p* < 0.01, ^∗∗∗^*p* < 0.001 (*n* = 10).

We first examined the dose-effect relationship and pharmacodynamic properties of supaglutide in normal ICR mice. We found that a single subcutaneous injection with different doses of supaglutide significantly decreased glucose excursion in response to an oral glucose challenge in a dose-dependent fashion (Figure 2). As shown, while it did not obviously affect the fasting blood glucose levels, the hypoglycemic effect was observed with statistical significance during an OGTT conducted at 48 h (0.15 mg/kg, *p* < 0.001) and 72 h (0.3 mg/kg, *p* < 0.001) after a single injection of supaglutide (Figures 2A–C). Notably, the high dose group (0.6 mg/kg) yet showed noticeable glucose-lowering effect during an OGTT at 96 h, though without statistical significance (Figure 2D, *p* = 0.08).

### Supaglutide Lowers Blood Glucose and Increases Insulin Secretion in Hyperglycemic *db/db* Mice

**FIGURE 3:**
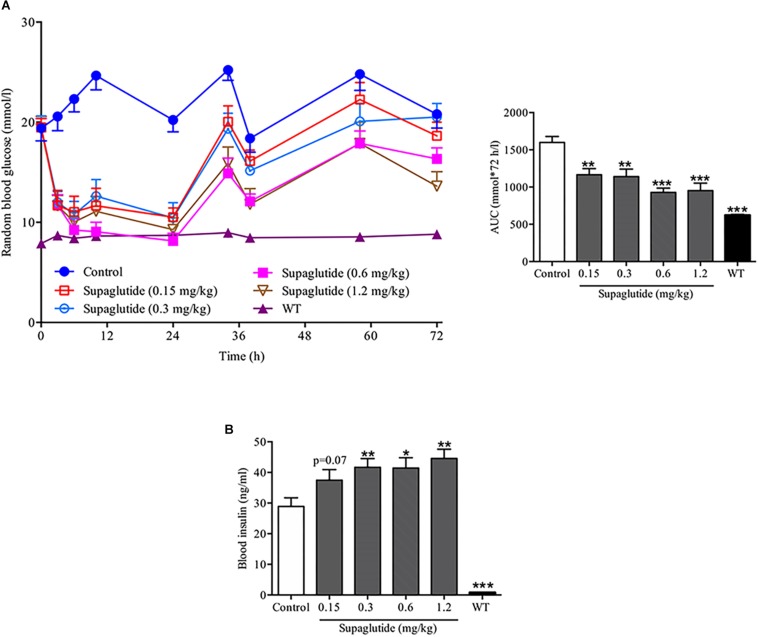
A single subcutaneous injection of supaglutide significantly reduces blood glucose and increases plasma insulin levels in *db/db* mice. **(A)** Random blood glucose levels and the area under the curve (AUC_0–72__h_). **(B)** The level of blood insulin was measured at 3 h after a single subcutaneous injection. Data are shown as mean ± SEM. ^*^p < 0.05, ^∗∗^*p* < 0.01, ^∗∗∗^*p* < 0.001 (*n* = 10).

To further investigate the anti-diabetic effects of supaglutide, the random blood glucose levels within 72 h were measured after the first injection in *db/db* mice. All supaglutide treated mice showed significant glucose-lowering effect (Figure 3A). Moreover, the duration of glucose-lowering effect was dose-dependent (Figure 3A). Specifically, at 72 h, the groups of mice received 0.6 and 1.2 mg/kg maintained reduction in blood glucose of 21.4 and 34.5% compared to the mice received placebo injection (*p* < 0.05 and *p* < 0.001).

To examine the insulinotropic effects of supaglutide in *db/db* mice, blood samples were collected at 3 h after the first drug administration, serum was then separated and measured for insulin levels. It showed that while the insulin levels in 0.15 mg/kg supaglutide group increased by 29.7% (*P* = 0.07), plasma insulin levels in 0.3, 0.6, and 1.2 mg/kg supaglutide mice were significantly higher than those in control group (Figure 3B). These data illustrated that supaglutide significantly decreased hyperglycemia which was associated with elevation of insulin secretion after a single subcutaneous injection in diabetic *db/db* mice.

### Supaglutide Reduces Blood Frutosamine Levels in *db/db* Mice With Multiple Subcutaneous Injections

**FIGURE 4:**
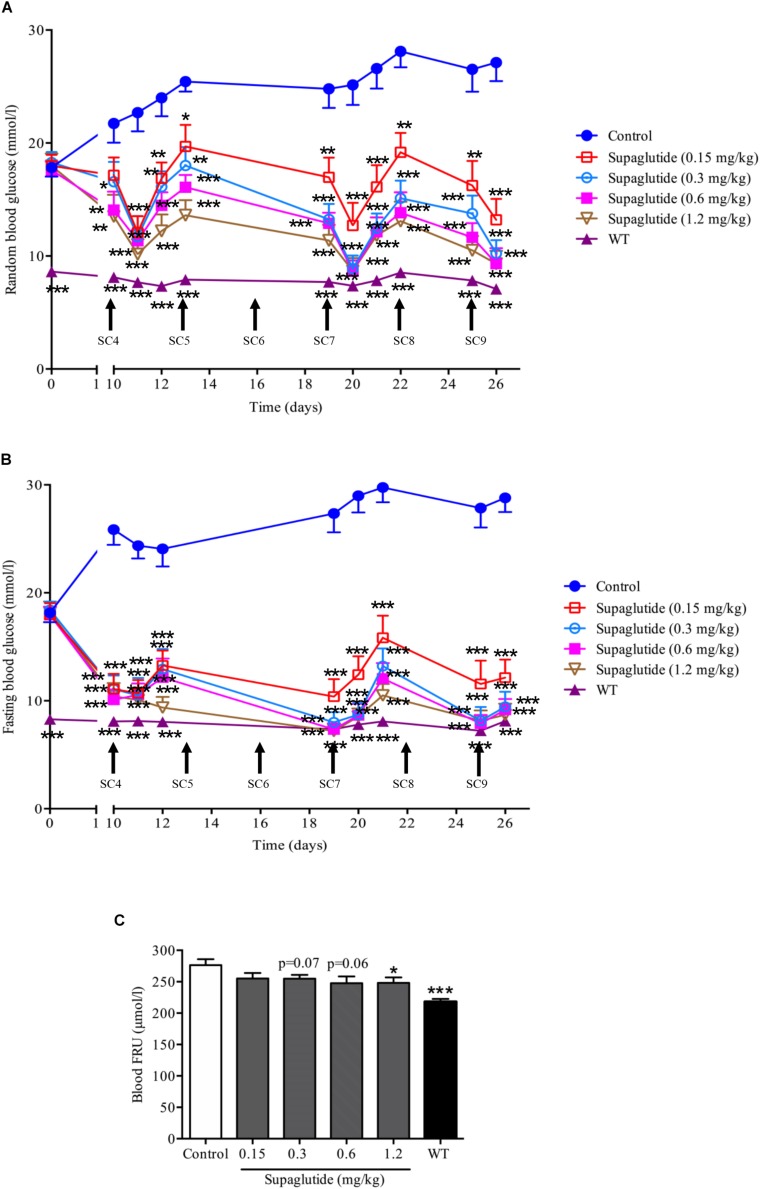
Long-term treatment with supaglutide dose-dependently reduces random blood glucose, fasting blood glucose and fructosamine (FRU) levels in *db/db* mice. **(A)** Random blood glucose levels (shown from fourth SC). SC, subcutaneous. **(B)** Fasting blood glucose (shown from fourth SC). **(C)** Blood FRU level. Data are shown as mean ± SEM. ^*^p < 0.05, ^∗∗^*p* < 0.01, ^∗∗∗^*p* < 0.001 (*n* = 10).

To further investigate long-term glucoregulatory effects of supaglutide in *db/db* mice, subcutaneous injections at 0.15, 0.3, 0.6, and 1.2 mg/kg doses were conducted twice a week for 4 weeks. During treatment period, the blood glucose in non-treated *db/db* mice remained at a high levels and developed severe hyperglycemia (Figures 4A,B). In a contrast, supaglutide-treated *db/db* mice showed significantly reduced random (Figure 4A) and fasting (Figure 4B) blood glucose. The reduction in blood glucose appeared in a dose-dependent fashion. Particularly, the fasting blood glucose of mice with higher dose-injections (i.e., 0.6 and 1.2 mg/kg) reached to levels which were nearly closed to the wild type control mice (Figure 4B). Notably, the results revealed that the multiple subcutaneous injections of supaglutide (e.g., after three injections) dose-dependently produced a therapeutic glucose homeostasis (Figures 4A,B). Serum levels of fructosamine (FRU), a marker that reflects the average levels of blood glucose control over the past 2–3 weeks was measured at the end of the intervention (Figure 4C). Compared with the control group, the FRU level was decreased after supaglutide treatment. The FRU levels in 1.2 mg/kg supaglutide treatment mice was significantly lower than that in non-treated control group (*P* < 0.05), while 0.3 and 0.6 mg/kg supaglutide also showed a downward trend (*p* = 0.07, *p* = 0.06) (Figure 4C).

In addition, we found that supaglutide treatment significantly and dose-dependently improved glucose tolerance in *db/db* mice (Supplementary Figures 2A,B) and increased insulin secretion stimulated by glucose (Supplementary Figures 2C,D).

### Long-Term Supaglutide Treatment Enlarges Beta-Cell Mass and Increases Plasma Insulin Levels in *db/db* Mice

**FIGURE 5:**
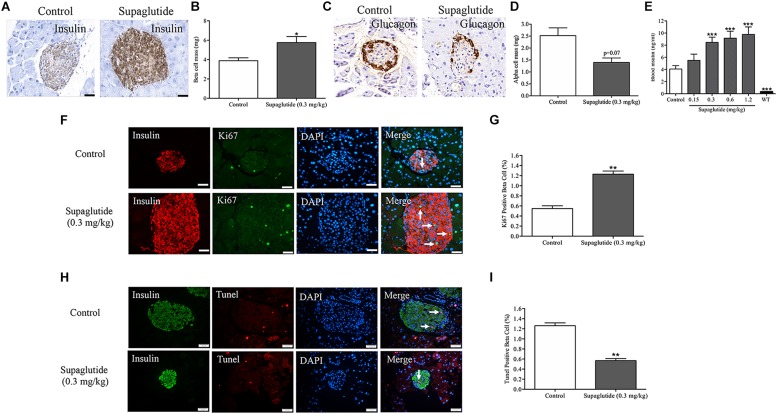
Long-term treatment with supaglutide improves beta-cell mass and increases plasma insulin level in *db/db* mice. **(A)** Staining of insulin (brown) in pancreatic sections of non-treated *db/db* mice and supaglutide (0.3 mg/kg) treated mice. **(B)** Beta-cell mass. **(C)** Staining of glucagon (brown) in pancreatic sections of non-treated *db/db* mice and supaglutide (0.3 mg/kg) treated mice. **(D)** Alpha-cell mass. **(E)** Blood insulin levels. **(F)** Double staining of islets, showing Tunel+ (green) and insulin+ (red) cells. **(G)** Quantitation of Tunel+ beta-cells. **(H)** Double staining of islets, showing Ki67+ (green) and insulin+ (red) cells. **(I)** Quantitation of Ki67+ beta-cells. Data are shown as mean ± SEM. ^*^p < 0.05, ^∗∗^*p* < 0.01, ^∗∗∗^*p* < 0.001 (*n* = 10).

In rodents, GLP-1 exerts anti-diabetic effects which were associated with increased beta-cell proliferation and increased beta-cell mass (Wang and Brubaker, 2002; Drucker and Nauck, 2006). We then further analyzed beta-cell mass and plasma insulin level at end of the long-term supaglutide treatment. Our results showed that beta-cell mass were significantly increased in supaglutide treated mice (0.3 mg/kg) compared to the non-treated control mice (Figures 5A,B). This was associated with decreased alpha-cell mass in supaglutide-treated db/db mice (Figures 5C,D). Supaglutide-treated diabetic mice showed increased plasma insulin levels in a dose-dependent manner after 4-weeks treatment (Figure 5E), suggesting that supaglutide-induced increases in the beta cell mass that was functionally relevant. We examined the effects of supaglutide in the beta-cell proliferation and survival as determined by the ki67-insulin and/or Tunel-insulin double staining in the pancreatic sections. The results showed that supaglutide treatment increased Ki67 positive beta-cells (Figures 5F,G), and decreased Tunel positive beta-cells (Figures 5H,I), suggesting its potential mechanism underlying the regulation of beta cell mass in diabetic mice.

### Supaglutide Reduces Body Weight Gain in Obese *db/db* Mice

**FIGURE 6:**
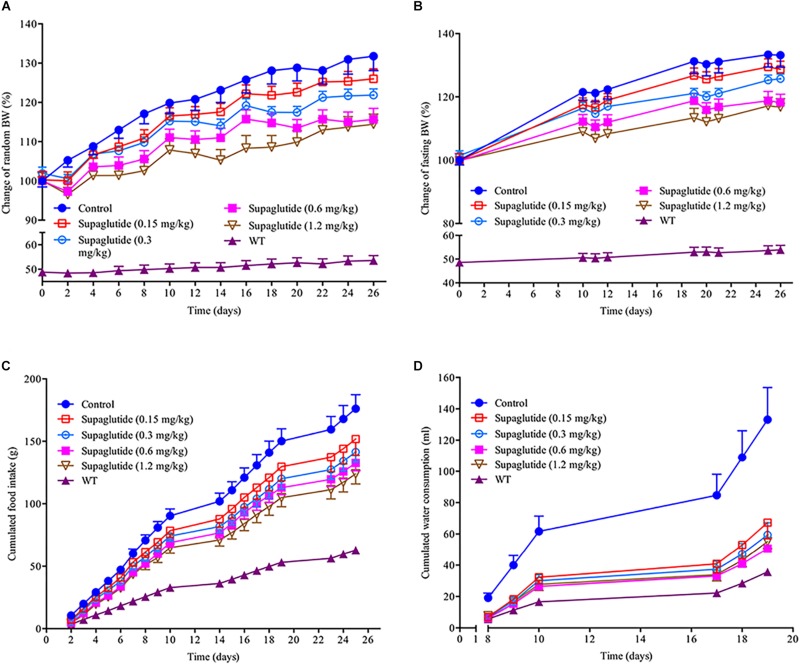
Long-term treatment with supaglutide reduces body weight, food intake and water consumption in *db/db* mice. **(A)** Random body weight. **(B)** Fasting body weight. **(C)** The cumulated food intake. **(D)** The cumulated water consumption. Data are shown as mean ± SEM. ^*^p < 0.05, ^∗∗^*p* < 0.01, ^∗∗∗^*p* < 0.001 (*n* = 10).

Changes in body weight, food intake and water consumption were monitored in *db/db* mice at indicated time points throughout the experiment. As shown, the treatment with supaglutide significantly decreased both random and fasting body weight gain in a dose dependent manner compared with non-treated *db/db* mice (Figures 6A,B). The non-treated *db/db* mice showed polyphagia and polydipsia compared to non-diabetic mice. However, these diabetic symptoms were significantly improved in supaglutide-treated *db/db* mice as determined by the accumulated food and water consumption (Figures 6C,D).

We also found that the weight sparing effect of supaglutide was related to the reduction of the white fat, especially epididymal fat and inguinal fat (Table 1). The weight of epididymal fat were significantly reduced at all doses supaglutide treated mice compared to non-treated *db/db* mice. The inguinal fat weight was significant decreased in 0.6 and 1.2 mg/kg supaglutide treated mice. However, the weight of brown fat was not significantly changed (Table 1).

**TABLE 1:** Effects of supaglutide on fat tissue weight in *db/db* mice.

**Tissue weight (g)**	**WT**	**Control**	**Supaglutide** **(0.15 mg/kg)**	**Supaglutide** **(0.3 mg/kg)**	**Supaglutide** **(0.6 mg/kg)**	**Supaglutide** **(1.2 mg/kg)**
Epididymal fat	0.009 ± 0.002^*⁣**^	0.058 ± 0.004	0.039 ± 0.002^**^	0.041 ± 0.004^*^	0.037 ± 0.003^*⁣**^	0.033 ± 0.003^**^
Inguinal fat	0.138 ± 0.008^*⁣**^	3.560 ± 0.170	3.082 ± 0.154	2.924 ± 0.163	2.863 ± 0.140^**^	2.700 ± 0.150^**^
BAT	0.065 ± 0.004^*⁣**^	0.264 ± 0.014	0.225 ± 0.011	0.227 ± 0.011	0.223 ± 0.009	0.227 ± 0.017

Data are shown as mean ± SEM. ^*^p < 0.05, ^∗∗^*p* < 0.01, ^∗∗∗^*p* < 0.001. Epididymal fat (*n* = 5), the other tissue weight (*n* = 10).

Using TD-NMR based technology we measured fat tissue, lean tissue and body fluid in the mice on day 24 after drug administration. Table 2 summarizes the percentage of fat weight, muscle weight and body fluid to the body weight. Compared with non-treated *db/db* mice, mice received supaglutide injections showed no significant changes in body fluid, but reduced fat weight and increased muscle weight, suggesting that long-term supaglutide therapy exerts weight sparing effect which is mainly due to the reduced fat mass (but not muscle mass) (Table 2).

**TABLE 2:** Body composition analysis in *db/db* mice.

**Group**	**Day 0**			**Day 24**		
		
	**Fat**	**Muscle**	**Body fluid**	**Fat**	**Muscle**	**Body fluid**
WT	3.2 ± 0.6^*⁣**^	80.3 ± 0.6^*⁣**^	7.7 ± 0.5	3.3 ± 0.4^*⁣**^	77.8 ± 0.5^*⁣**^	6.3 ± 0.4
Control	42.5 ± 1.1	46.7 ± 0.8	8.0 ± 0.2	57.3 ± 0.9	33.6 ± 1.0	6.7 ± 0.3
Supaglutide (0.15 mg/kg)	41.6 ± 1.0	47.9 ± 0.8	7.8 ± 0.2	54.5 ± 1.4	35.5 ± 1.0	6.1 ± 0.4
Supaglutide (0.3 mg/kg)	44.2 ± 1.2	45.8 ± 1.0	7.7 ± 0.3	54.0 ± 1.1^*^	36.3 ± 0.9	6.5 ± 0.2
Supaglutide (0.6 mg/kg)	43.4 ± 1.2	46.3 ± 0.8	7.6 ± 0.2	54.5 ± 1.4	36.6 ± 1.0	6.2 ± 0.4
(*P* = 0.05)						
Supaglutide (1.2 mg/kg)	44.9 ± 1.2	47.5 ± 1.0	8.1 ± 0.3	51.3 ± 1.4^**^	38.7 ± 1.2^**^	6.9 ± 0.4

Data are shown as mean ± SEM. ^*^p < 0.05, ^∗∗^*p* < 0.01, ^∗∗∗^*p* < 0.001 (*n* = 10).

### Long-Term Supaglutide Treatment Improves Lipid Profile in *db/db* Mice

**FIGURE 7:**
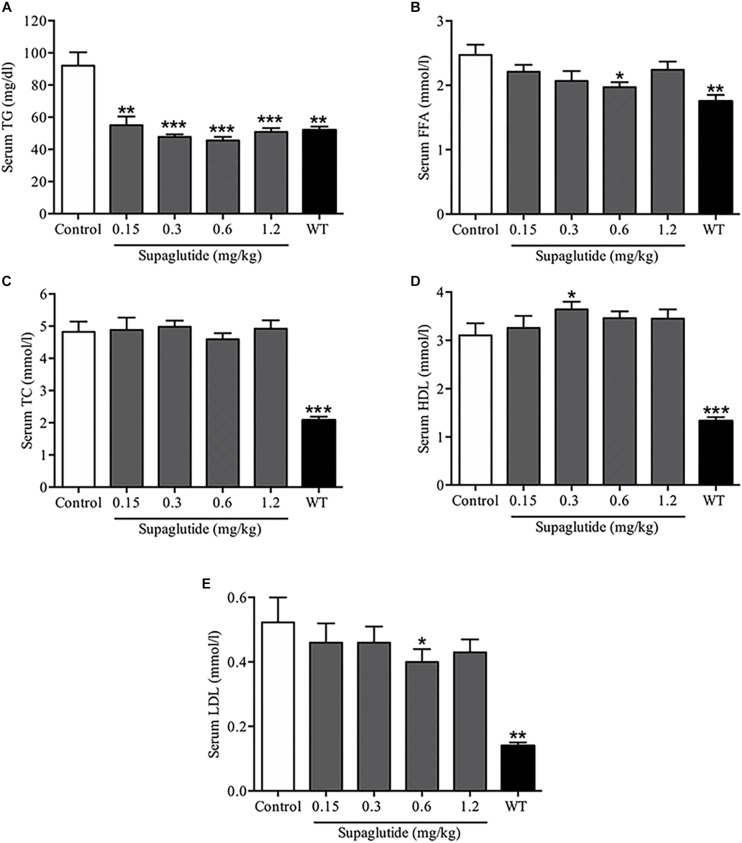
Long-term treatment with supaglutide improves lipid profile in *db/db* mice. **(A)** The level of serum triglyceride (TG). **(B)** The serum free fatty acid (FFA) levels. **(C)** Total cholesterol (TC) levels. **(D)** High-density lipoprotein (HDL) levels. **(E)** Low-density lipoprotein (LDL) levels. Data are shown as mean ± SEM. ^*^p < 0.05, ^∗∗^*p* < 0.01, ^∗∗∗^*p* < 0.001 (*n* = 10).

Serological analysis exhibited that supaglutide treatment dramatically decreased serum TG levels (Figure 7A). The serum FFA levels were significantly lowered in supaglutide treatment group (0.6 mg/kg) compared to the non-treated *db/db* mice (Figure 7B). Interestingly, supaglutide treatment did not significantly change the levels of serum TC (Figure 7C). The HDL levels in 0.3 mg/kg supaglutide treatment mice was significantly higher than that in non-treated db/db group (*P* < 0.05), while other dose of supaglutide also showed a upward trend (Figure 7D). The LDL level in 0.6 mg/kg supaglutide treated mice was significantly lower than that in non-treated db/db group (*P* < 0.05), while other dose of supaglutide treated mice showed a downward trend (Figure 7E).

## Discussion

The present study shows that supaglutide dose-dependently stimulates insulin secretion in both mouse and human islets, and that supaglutide therapy over prolonged periods of time (twice a week injection for 4-weeks) results in an overall improvement in glycemic control, amelioration of obesity and improving lipid metabolism in hyperglycemic obese *db/db* mice.

Therapy targeting GLP-1 signaling has emerged as alternatives to basal insulin for treatment intensification in patients inadequately controlled with oral antidiabetic drugs. Importantly, GLP-1 exerts insulinotropic effects in a glucose-dependent fashion (Meloni et al., 2013; Nadkarni et al., 2014). The glucose level-dependent hypoglycemic characteristics of GLP-1 are responsible for the very low incidence of hypoglycemia observed during the clinical treatment of type 2 diabetes mellitus (Drucker, 2006; Trujillo and Nuffer, 2014).

Our *in vitro* studies showed that supaglutide drastically and dose-dependently stimulated insulin secretion in isolated mouse and human islets in the presence of low glucose (2 mM) and/or, of high glucose (16.7 mM). Observations that GLP-1 enhanced insulin secretion under low glucose conditions were also reported by others studies, using either isolated islets or whole pancreas perfusion technique (Fridolf et al., 1991; Goke et al., 1993; Meloni et al., 2013). Indeed, increases in basal insulin levels in the absence of elevated blood glucose was also found in fasted humans (Kreymann et al., 1987) and mice (Fridolf et al., 1991). These observations may provide the major consequences to understand the physiological role of GLP-1 in the enteroinsular axis, consisting not only incretin and insulin, but also other factors such as glucagon or other agents (e.g., γ-aminobutyric acid, GABA) (Flatt et al., 2009; Wang and Jin, 2009). Glucagon, the insulin counter regulatory hormones plays an important role in preventing hypoglycemia, particularly when the body receives an insulin-stimulating agent under fasting conditions (Li et al., 2015). In this circumstance, an insulin-stimulating agent increases circulating levels of insulin and glucagon in healthy human subjects under fasting conditions (the ratio of insulin to glucagon is unchanged), allowing body to maintain to maintain body euglycemia (Li et al., 2015; Kalra and Gupta, 2016). Such the fine-tuning mechanism in maintaining blood glucose homeostasis is also found in the islets to prevent a potential hypoglycemia resulting from insulin “overshooting” (Dong et al., 2006; Bansal and Wang, 2008). In accord with this concept, it is not surprising to see that under *in vitro* conditions GLP-1 not only enhances insulin secretion, but also stimulates glucagon release by activation of GLP-1 receptor mediated signaling pathway involving protein kinase A and opening the voltage-dependent Ca^2+^ channels (Ding et al., 1997; Zhang et al., 2019). In isolated human islets, in the presence of high glucose (16.7 mM), supaglutide increased insulin secretion by sixfold compared with that at low glucose (2 mM), indicating extensive capacity in the stimulation of insulin secretion.

We examined the long-term glyco-regulatory effect of supaglutide in *db/db* mice. The results showed that the blood glucose levels were decreased rapidly after the first injection. The decrease in blood glucose levels were in a dose-dependent manner and tended to be stable after 2–3 injections of supaglutide. The therapy obviously improved diabetic symptoms, exemplified by significantly decreased water consumption in *db/db* mice on supaglutide therapy. These findings are consistent with clinical observations that polydipsia and polyuria associated with persistent hyperglycemia in diabetic patients and improvement in polydipsia and polyuria are the result of ameliorated diabetic hyperglycemia or improved glucose tolerance (Park et al., 2016). Of note, the supaglutide-treated mice showed enlarged beta-cell mass, increased pancreatic insulin content, and elevated circulating insulin levels, suggesting the enhanced functional beta-cell may represent a cellular mechanism underlying the improved glycemia control and diabetic symptom in diabetic mice. GLP-1 stimulates beta-cell mass expansion by activating proliferation and inhibiting apoptosis signaling pathways (Wang and Brubaker, 2002; Lee and Jun, 2014), and enhancing functional beta-cell mass is recently considered as a potential therapeutic target for diabetes (Chen et al., 2017).

Furthermore, the long-term supaglutide treatment significantly reduced food intake associated with decreased body weight gain in these obese mice. It is conceivable that the effects of GLP-1 on suppression of appetite and satiety, as well as the slowing gastric emptying appear to be a major contributor to the reduction of food intake and subsequent weight loss (Shah and Vella, 2014). It is interesting to note, our previous studies (Wang and Brubaker, 2002), consistent with others earlier studies (Greig et al., 1999), showed that exendin-4 once daily injection for two weeks, significantly improved glycemic control, but had no significant effect on body weight gain in diabetic db/db mice. This suggests that GLP-1’s insulinotripic and/or hypoglycemic effects can be separated from its weight sparing effects. GLP-1Rs are expressed on hypothalamic neurons (Turton et al., 1996), and endogenous GLP-1 appears to involve in postprandial satiating effects in the CNS (Heppner and Perez-Tilve, 2015; ten Kulve et al., 2015). Although the blood-brain barrier is relatively impermeable to larger proteins under both normal physiological conditions and in the setting of diabetes (Bickel et al., 2001), a previous study suggested that a GLP-1/albumin fusion molecule exhibited anorectic effects after peripheral administration (Baggio et al., 2004). The ability of supaglutide to penetrate the blood-brain barrier needs to be further explored.

In this study, we observed that supagutide reduced obesity in *db/db* mice which was associated with fat loss and muscle gain, further suggesting that the adipose may be a direct therapeutic target. Evidence support this notion is that our previous studies in established obese mice with high-fat diet (HFD) feeding showed that supaglutide exerted body weight sparing effects which was associated with enhanced brown remodeling of white adipose tissue (Wan et al., 2017). Specifically, supaglutide treatment upregulated Ucp1, a protein that is responsible for brown fat thermogenesis (Feldmann et al., 2009), and increased tolerance of the HFD-mice to cold environment, suggestive of enhanced energy expenditure (Wan et al., 2017).

Supaglutide therapy improved metabolic profile in *db/db* mice, exemplified by significant reduction in TG, FFA, and LDL, and increased HDL, which is in agreement with our previous studies demonstrating that supaglutide significantly improved lipid metabolism in HFD-induced obese mice (Wan et al., 2017). Diabetic dyslipidemia represents the major link between diabetes and the increased cardiovascular risk of diabetic patients (Mokdad et al., 2003; Xiong et al., 2013). Type 2 diabetic *db/db* mice develop heart dysfunction which is associated with elevated TG serum levels in an age-dependent manner (Dludla et al., 2017). Long-term supaglutide therapy resulted in reduced hyperglycemia, ameliorated obesity and improved lipid metabolism implying its anti-diabetic cardiac benefits, in accord with the clinical evidence that GLP-1 therapy revealed beneficial effects in cardiac dysfunction (Du et al., 2016; Marso et al., 2016a,b). Of note, a 4-weeks supaglutide intervention did not affect the TC levels in the *db/db* mice, which is inconsistent with other’s studies demonstrated that prolonged treatment of GLP-1 receptor agonists GLP-1 resulted in an obvious TC levels decline in *db/db* mice (Zhang et al., 2015; Han et al., 2018; Patel et al., 2018).

A therapy targeting GLP-1 signaling produces hypoglycemic and weight sparing effects offering therapeutic benefits to diabetes, since the majority of T2D patients are obese, the further weight gain associated with anti-diabetic therapy (e.g., insulin injections) may deteriorate metabolic consequence and increasing risk of death from the disease-related complications including microvascular and macrovascular complications (Calle et al., 1999). Clinical studies demonstrated that GLP-1 therapies are associated with fewer hypoglycemic events than insulin, with weight sparing and cardiovascular beneficial effects (Heo et al., 2012; Li et al., 2012; Diamant et al., 2014; Ostergaard et al., 2017). Given its long-lasting GLP-1 actions and potential clinical compliance, it is of great interests to investigate the molecular mechanism of supaglutide in regulating lipid metabolism and exerting cardioprotective effects in both preclinical and clinical settings.

In a summary, our results suggest that supaglutide retains insulinotropic property, exerts long-lasting hypoglycemic effects through enhancing functional beta-cell mass, and exerts anti-obesity effects through reducing energy intake and enhancing energy expenditure. Its anti-diabetes and obesity effects with improved lipid metabolism provide supaglutide as an alternative new tool to elucidate GLP-1 biology, seeking novel therapeutic targets for treating diabetes, obesity and its associated metabolic disorders.

## Data Availability

The datasets generated for this study are available on request to the corresponding author.

## Ethics Statement

All animal experiments followed the National Institute of Health Guidelines on the Care and Use of Animals and were approved by the Institutional Animal Care and Utilization Committee (IACUC), Shanghai Institute of Materia Medica, Chinese Academy of Sciences. The human experiments was reviewed and approved by the University Health Network Research Ethics Board (14-8321.2) (University of Toronto, Toronto, ON, Canada). The subject gave written informed consent in accordance with the Declaration of Helsinki.

## Author Contributions

QW and YLe contributed to the conception and design of the study. WL, YF, and LZ contributed to performing the major body of the experiments. LR, QC, and WL analyzed the data. WS, YY, ZZ, YLi, TJ, and GP provided the reagents and technique advice. QW, LR, and YLi contributed to writing of the manuscript. All authors reviewed and approved the manuscript.

## Conflict of Interest Statement

QW has GLP-1 related patents. The remaining authors declare that the research was conducted in the absence of any commercial or financial relationships that could be construed as a potential conflict of interest.
