# Supplementary material for: Novel GLP-1 Analog Supaglutide Stimulates Insulin Secretion in Mouse and Human Islet Beta-Cells and Improves Glucose Homeostasis in Diabetic Mice
Source: Front Physiol. 2019 Jul 25;10:930. doi: 10.3389/fphys.2019.00930 (PMC6670290; doi:10.3389/fphys.2019.00930)
Supplement: Supplementary file 1 [file Data_Sheet_1.PDF]

Supplemental figure 1. The microscopic images of isolated mice and human islets. (A) The images of isolated mice islets. (B) The viability of human islets morphology was determined (left panel), and glucose-stimulated insulin secretion assays were performed to check the secretory function (right panel). LG: 2 mM, HG: 16.8 mM.

Supplemental figure 2. Long-term treatment with supaglutide increased insulin secretion. (A) IPGTT was performed after long-term treatment with supaglutide. Blood glucose levels were measured at indicated time points after 1 g/kg glucose injection, (B) The areas under the glycemic curves (AUC). (C) Glucose stimulated insulin secretion (GSIS) assay was performed during IPGTT. Blood insulin levels were measured at indicated time points after 1 g/kg glucose injection. (D) The areas under the glycemic curves (AUC). *$p < 0.05$ versus control, **$p < 0.01$ versus control, ***$p < 0.001$ versus control.
