# Supplementary figures and images for: Novel GLP-1 Analog Supaglutide Stimulates Insulin Secretion in Mouse and Human Islet Beta-Cells and Improves Glucose Homeostasis in Diabetic Mice
Source: Front Physiol. 2019 Jul 25;10:930. doi: 10.3389/fphys.2019.00930 (PMC6670290; doi:10.3389/fphys.2019.00930)

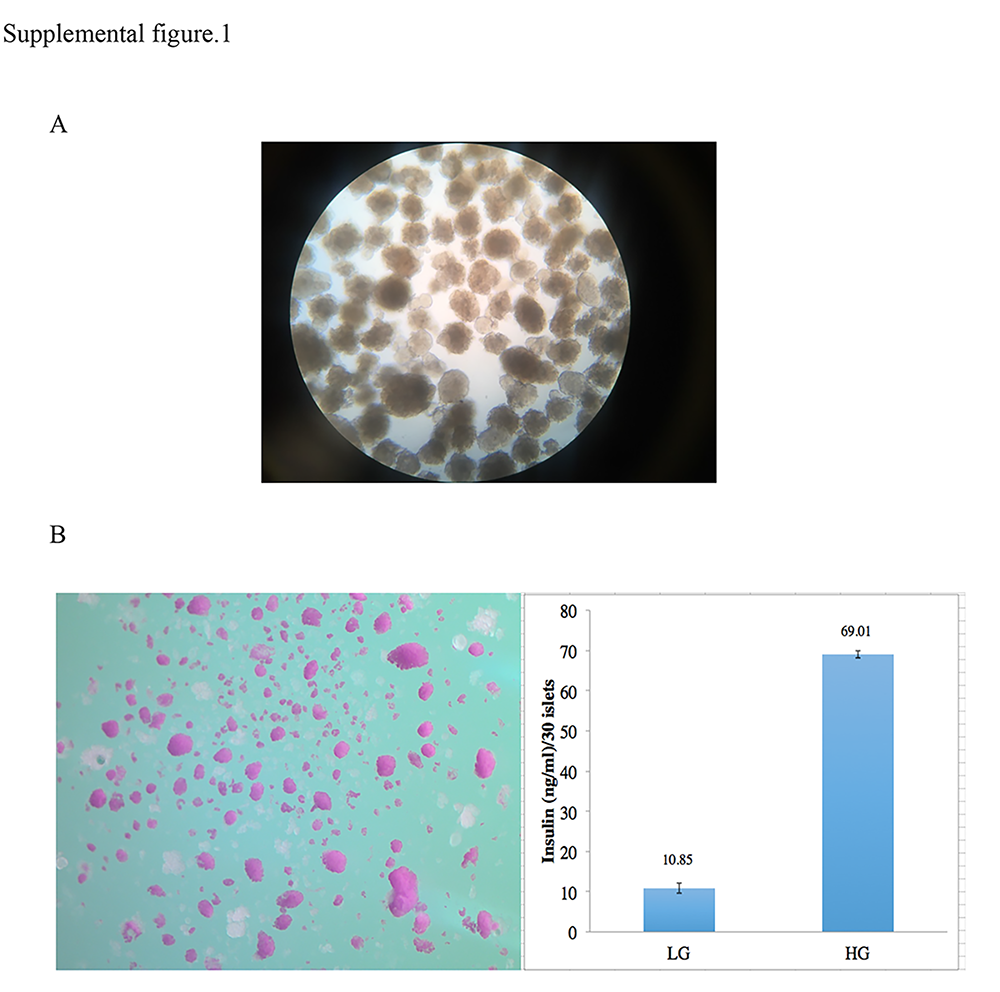

Supplement: Supplementary file 2 [file Image_1.TIF]

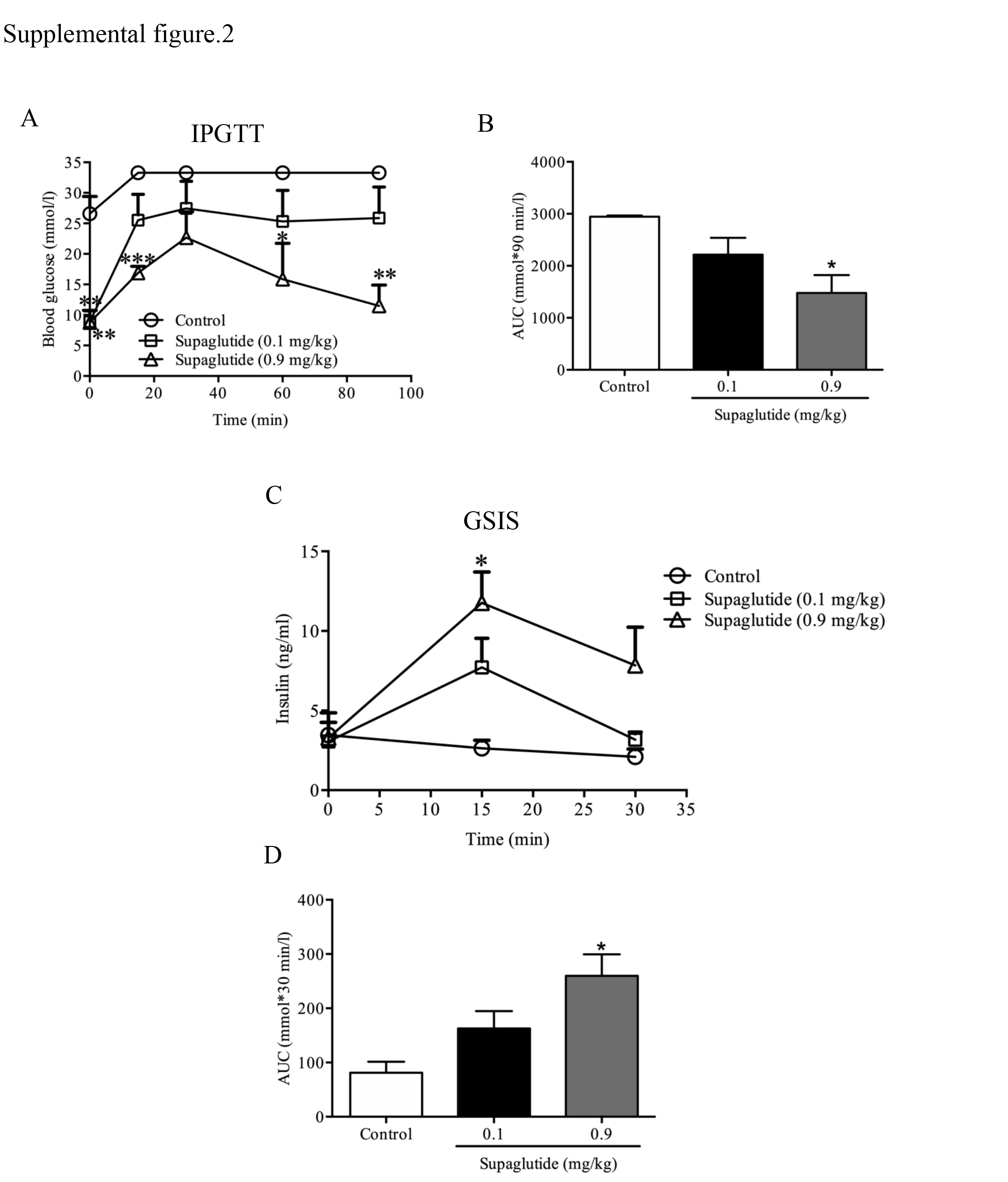

Supplement: Supplementary file 3 [file Image_2.TIF]
